# Appropriate and inappropriate care in the last phase of life: an explorative study among patients and relatives

**DOI:** 10.1186/s12913-016-1879-3

**Published:** 2016-11-15

**Authors:** Eva Elizabeth Bolt, H Roeline Willemijn Pasman, Dick Willems, Bregje Dorien Onwuteaka-Philipsen

**Affiliations:** 1 Department of Public and Occupational Health, EMGO Institute for Health and Care Research, VUmc Expertise Center for Palliative Care, VU University Medical Center, Van der Boechorstraat 7, 1081 BT Amsterdam, The Netherlands; 2 Department of General Practice, Section of Medical Ethics, Academic Medical Center, University of Amsterdam, Amsterdam, The Netherlands

**Keywords:** End-of-life care, Appropriate care, Patient perspective, Quality of care, Palliative care

## Abstract

**Background:**

Many people are in need of care in the last phase of life. However, the care they receive is not always appropriate. For instance, people can receive overly aggressive treatment or can have limited access to palliative care. The term appropriate care is often used by policy makers, while it is unclear what care recipients consider as appropriate care. This study aims to identify what care patients and relatives perceive as appropriate and as inappropriate in the last phase of life, for patients suffering from different conditions.

**Methods:**

We designed an online survey with open questions. Participants were recruited through organizations for patients, older people and medical professionals. Answers were analysed after data-driven coding. Forty-five patients and 547 relatives described the care they received and described why this care was appropriate or inappropriate.

**Results:**

Participants described more cases of appropriate care than inappropriate care. The cases of appropriate care were diverse, but all involved care in (one or more of) five dimensions; supportive care, treatment decisions, location, the role of the patient’s wish and communication. Each of these dimensions was frequently described (39-62 %). When care was inappropriate, this mostly involved inappropriate treatment decisions (69 %; especially overtreatment was frequently mentioned), and poor communication (50 %). There was considerable consistency in what was seen as (in)appropriate care across different conditions. However, especially patients suffering from other physical diseases than cancer more often received inappropriate care.

**Conclusion:**

From the perspective of patients and relatives, appropriate care in the last phase of life is a broad concept. Caregivers should be aware of the diversity of care needs in the last phase of life. Especially treatment decisions and communication can be improved.

## Background

*“Despite all, it became a rich time for her family and friends, and most of all [the effect of the care was that] my friend herself could complete her life in peace.*”*(participant about her friend with terminal cancer)*

Providing care in the last phase of life is a rewarding, yet challenging task. People at the end of life often have diverse physical, psychological, social and spiritual needs, as well as a need to prepare for death and achieve life closure [1–3]. But while patients and relatives attach great value to fulfilling these needs, [2] there is often hope for cure or life-prolongation at the same time [4]. Consequently, care often focusses on multiple goals simultaneously; palliation, life-prolongation and even cure. Unfortunately these aims are not always compatible; care aimed at cure or life-prolongation generally reduces quality of life in the short term. Despite this, the use of aggressive treatment at the end of life is increasing in western countries [5, 6]. At the same time, care can also be insufficient in fulfilling the patient’s needs. For instance, some groups of patients may have limited access to palliative care or receive lower quality curative care [7–9].

A term that is increasingly used in end-of-life care is appropriate care [10–12]. When the term is used by policy makers or medical organizations it refers to evidence-based and cost-effective care, that is aimed at improving the patient’s quality of life and is consistent with his or her preferences [13–15]. Although the patient’s perspective is an important aspect of appropriate care, it is not described how patients interpret this term. What do they describe as appropriate care in the last phase of life, and when do they speak of inappropriate care? Studies on patients’ and relatives’ perceptions of care at the end of life have mostly been limited to quality of palliative (cancer) care, identifying important elements of care such as communication, decision making, accessibility of care, symptom control and attention for psychological and social needs [3, 16]. A review showed that patients with and without cancer seem to suffer from similar problems in the last phase of life, [17] even though the trajectories of decline differ [18]. Does this mean that appropriate care is similar in different disease groups as well?

This study aims to: 1) determine what care patients and relatives perceive as appropriate and as inappropriate in the last phase of life; 2) to describe which patient characteristics and care characteristics are associated with care being inappropriate; and 3) to describe whether perspectives on (in)appropriate care differ for patients with cancer, other physical diseases, general decline and dementia.

## Methods

### Design

This study was performed among people who were in their last phase of life, and their relatives. Because there is no database representing these people, random sampling was not considered possible. To reach people in different situations, we recruited participants through patient organizations, medical organizations and an organization for older people. The last phase of life is not clearly defined in the literature. Therefore a broad definition was used, derived from the description of end of life by the National Institutes of Health (USA); ‘a phase in which someone suffers from a severe incurable disease and/or is at high age and requires care’ [19].

### Participants

Potential participants were invited to participate by the different organizations through e-mail, newsletters, social media and websites. Participating patient organizations were the Federation of Patients and Consumer Organisations in the Netherlands (NPCF), the Dutch Patient Organization (NPV), the Dutch Federation of Cancer Patient Organizations (NFK) and Patient Organization Blood cancer, Lymph cancer, Stem cell transplant (Hematon). Also, the largest Dutch organization for older people, Unie KBO (the Union of Catholic elderly unions), and one citizen organization, Right to Die-NL (an organization promoting autonomy in the last phase of life), participated. Participating organizations for medical professionals (who were asked to participate themselves or to invite patients to participate) were the Royal Dutch Medical Association (KNMG), Dutch Nurses’ Association (V&VN), national centre for palliative care (Agora) and Comprehensive Cancer Centre the Netherlands (IKNL). Participants who were in the last phase of life or who described care in the last phase of life of a relative/close friend were included. Exclusion criteria were: 1) Being involved in the described case as health care professional, and 2) cases in which the patient was not in the last phase of life (younger than 70 years and not suffering from a severe incurable disease).

### Questionnaire

An online questionnaire was designed especially for this study and tested by 37 patients, relatives and professional caregivers. The questionnaire was accompanied by an information sheet, no consent form was required because consent was implied by filling out the questionnaire. The term appropriate care was introduced by the following sentences: ‘*Good care is appropriate care. Not too much, not too little. Care that is in line with the patient’s needs and preferences.*’ The questionnaire started with the question: ‘*Did you or somebody close to you receive care in the last phase of life?’* The last phase of life was defined as having reached a high age or having a severe incurable disease. If the answer was yes, participants were invited to describe the care, and label it as appropriate or inappropriate. Participants were probed to elaborate on the case through four open questions: ‘*1) Can you describe the situation and the care received? 2) Why do you consider this care appropriate/inappropriate? 3) What was the consequence of the appropriate/inappropriate care? 4) Can you describe what you think has caused or contributed to the appropriate/inappropriate care?’.* Also, closed questions were included on participant, patient and care characteristics.

### Data analysis

The answers to the four open questions were coded using a data-driven approach (the codes were derived from the data instead of being determined beforehand). The codes were divided into three groups: 1) description of the care, 2) perceived causes, and 3) consequences. For this article only the codes in group 1 were used. The main researcher developed a coding scheme on the basis of the first 100 cases of appropriate care. Thereafter, another hundred cases were coded by both the main researcher and a medical student independently and their coding was compared. Any disagreement was discussed before they continued individually. Moreover, when there was doubt or new codes were identified during the process, this was discussed. Extra care was taken to ensure that only those aspects of care that were explicitly described as appropriate were coded. For instance, if a participant described care that was situated at home, the category ‘home’ was only coded if the participant described that being home was appropriate or inappropriate. For the cases of appropriate care we coded 21 categories. Five overarching care dimensions were defined. The process was repeated for cases of inappropriate care, resulting in 20 categories. All but one category of inappropriate care (errors and complications, described in 4 % of cases) could be grouped into the same five dimensions defined in appropriate care. Table 1 describes the categories and dimensions. The categories and dimensions were imported into IBM SPSS Statistics software (Version 20.0) for further quantitative analysis. Missing values were excluded from analysis.

**Table 1:** Categories of (in)appropriate care

Dimensions/categories in appropriate care	Description of appropriate care (A)	Categories in inappropriate care^a^
Dimension 1: Supportive care	Care directed at support, helping the patient and relatives to cope with the situation and supporting him in his (everyday) needs	
1.1 Continuous support	The caregiver provides the patient with guidance and support, is available, stays in touch, anticipates and responds to changes.	1.1 Absence of A
1.2 Physical care	Sufficient/affectionate physical care by nurses or nursing aides.	1.2 Absence of A
1.3 Care for relatives	Formal caregivers provide sufficient care or support to relatives.	1.3 Absence of A
1.4 Psychosocial care	Care aimed at improving psychosocial wellbeing, such as care provided by psychologists and chaplains, support groups, and care which enables the patient to perform his social roles or to undertake pleasant activities.	1.4 Absence of A
1.5 Continuity and coordination	The involved caregivers work together and communicate, care is available and accessible.	1.5 Absence of A
1.6 Social support	Presence of informal care or support by relatives and acquaintances.	1.6 Absence of A
1.7 Other care aspects	Other supportive care, e.g. alternative medicine, physiotherapy.	-^c^
Dimension 2: Treatment decisions	Decisions made on treatment or other medical interventions, involving a physician	
2.1 Forgoing treatment	Forgoing or withdrawing treatment or diagnostic testing aimed at cure or life-prolongation.	2.1 Identical to A
2.2 Symptom control	Sufficient treatment aimed to prevent or reduce physical symptoms.	2.2 Absence of A
2.3 Assisted dying	Euthanasia or assisted dying, or the physician agrees to perform euthanasia or assisted dying if suffering were to become unbearable.	2.3 Refusal or postponing of A
2.4 Potentially curative/life-prolonging treatment	Treatment or diagnostic testing aimed at cure or life-prolongation.	2.4 Identical to A
Dimension 3: Location	The location of the patient (continuous or intermittent)	
3.1 Home	Being home (as much as possible) or going home.	3.1 Identical to A
3.2 Long-term care facility	Residing in a nursing home, residential home or hospice.	3.2 Identical to A
3.3 Hospital	Being admitted to a hospital or visiting a hospital (as outpatient or for emergency care).	3.3 Identical to A
3.4 Other location	Other location, e.g. psychiatric institution.	-^c^
Dimension 4: Role of the patient’s wish	Role of the patient’s wish in decision making	
4.1 Patient’s wish is followed	The patient’s wish is asked, expressed and/or followed (including following the patient’s advance care directive or relatives as surrogate decision maker).	4.1 Absence of A
4.2 Patient is in control	The patient maintains control over the situation (e.g. in medical decision-making, self-care).	4.2 Absence of A
Dimension 5: Communication	Patient-physician communication is sufficient	
5.1 Dialogue	The physician and patient (regularly) discuss future care (advance care planning) and make shared decisions.	5.1 Absence of A
5.2 Right attitude	The caregiver has a respectful, empathic or open attitude.	5.2 Absence of A
5.3 Being listened to	The caregiver shows interest in and listens to the patient.	5.3 Absence of A
5.4 Being informed	The patient and/or relatives are well informed (about the situation, prognosis, treatment options and side effects).	5.4 Absence of A
Other		
-^b^	-	6.1 Errors and complications

^a^The categories in inappropriate care were either the opposite of the categories in appropriate care (‘Absence of A’ or ‘Refusal or postponing of A’ or identical to the categories of appropriate care (‘Identical to A’)

^b^This category was not mentioned as appropriate care

^c^This category was not mentioned as inappropriate care

To study whether patient and care characteristics were associated with the occurrence of inappropriate care, the cases of appropriate and inappropriate care were compared. Compared characteristics were gender, age, diagnosis, location of care and the person(s) responsible for the described care. An association was assumed if the *p*-value was smaller than 0.05 in independent T-test for continuous variables, Fisher’s exact test for dichotomous variables and Chi-square (two-tailed) for categorical variables. By logistic regression analysis we analysed whether diagnosis was associated with prevalence of the five care dimensions. The studied diagnostic groups were cancer, other physical diseases, general decline/old age and dementia. Odds ratios were calculated for the association between the presence of each of these diagnostic groups separately and the prevalence in which the care dimensions were described, after correction for gender, age (categorized in four groups) and the presence of the other diagnostic groups (because more than one diagnosis could be present). The same was done for the separate treatment decisions, since these formed a heterogeneous group. We did not correct for multiple testing because that would lead to a high chance of type II errors. Instead, we report 95 % confidence intervals.

Because a large number of participants was recruited through the Right to Die-NL newsletter (44 %), we checked whether these participants differed from the other participants in logistic regression analysis. The same was done for participants (previously) working in health care (44 %), although in the described cases they were involved as patient or as relative. Some minor differences were found, which are described in the appendix.

## Results

### Participants

A total of 592 people participated; 45 patients (8 %) and 547 relatives (92 %). They described 429 cases of appropriate care and 309 cases of inappropriate care. In Table 2 the participant characteristics are shown. There were no significant differences between the participants describing appropriate care and those describing inappropriate care. Three-quarter of the described patients had died at the time of research.

**Table 2:** Participant characteristics^a^

	*n* = 592 %
Participant gender	
Female	67
Participant age	
mean (range)	60 (23-88)
18-49	15
50-64	47
65-79	33
80 and up	5
Ethnicity	
Ethnic minority^b^	6
Religion	
None	53
Christian	41
Other	7
(Former) health care worker	
Yes	44
The questionnaire was reached through:	
Right to Die-NL^c^	44
A colleague or acquaintance	21
Through social media/surfing, not further specified	12
Organizations for health care professionals^d^	8
Specific patient organizations^e^	5
Organization for older people^f^	5
General patient organizations^g^	4
Described case	
Appropriate care	48
Inappropriate care	28
Both	25
Relationship of patient to participant (total of 738 cases):^i^	
Participant is the patient	8
Parent (in law) of the participant	59
Partner	21
Brother/sister (in law)	9
Related, otherwise	7
Unrelated	6
Patient is deceased	74

^a^Missing values ranged from 0.0 to 1.5 %

^b^Participant is considered to be of an ethnic minority group if one or both parents are born outside the Netherlands

^c^Right to Die-NL: An organization that aims to enhance the autonomy and control of an individual when it comes to the last phase of life, focusing on euthanasia and (physician-)assisted suicide

^d^Agora, IKNL, KNMG, V&VN

^e^NFK and Hematon

^f^Unie KBO

^g^NPCF, NPV

^i^More than one answer possible: 11 % described two different cases

### What is appropriate and inappropriate care?

Table 1 shows the five dimensions and corresponding categories that are described in appropriate and inappropriate care. The five dimensions of appropriate care are supportive care, treatment decisions, location, role of the patient’s wish and communication. Most categories of inappropriate care were the direct opposite of categories in appropriate care (e.g. 1.1 ‘continuous support’ in appropriate care and 1.1 ‘absence of continuous support’ in inappropriate care), some categories were identified both in appropriate and in inappropriate care (e.g. 3.1 ‘home’). Table 3 shows some examples of case descriptions and corresponding categories.

**Table 3:** Some case descriptions. A few examples of case descriptions and coding. All names used are pseudonyms

Nr	Care	Characteristics	Care dimensions (categories)	Description
1	Appropriate	Male, 70-79 years, general decline, heart, lung and neurological disease, described by his daughter (40-49)	Supportive care (1.1, 1.3, 1.5, 1.6), Treatment decisions (2.1), Location (3.1), Patient’s wish (4.1).	While Mr Schoen was in the hospital for tests, he became increasingly confused. Therefore, his family decided to forgo further testing and bring him home. His wife and children were able to care for Mr Schoen, in close cooperation with a small team of nurses and the GP, who knew the patient well. The care was tailored to the family’s wishes, but the professional caregivers also intervened when necessary. The family felt supported and Mr Schoen died in a calm familiar setting.
2	Inappropriate	Female, 40-49 years, cancer, described by her brother (50-59)	Supportive care (1.1, 1.2, 1.3, 1.5), Communication (5.4).	Ms Kramer was discharged from the hospital knowing she would die soon. From that moment, it was unclear who was responsible for the care. Ms Kramer’s brother described: ‘We did not know how to take care of a dying person, what tools were available, what medicine we could give and how to get these.’ The home care sent different nurses every day, who did not know the situation. Their GP did not provide them with the information they needed. ‘At the same time, we did not know which questions we should have asked.’ After her death, her family was left with feelings of guilt because they felt Ms Kramer did not receive optimal care in her final days.
3	Inappropriate	Female, 70-79 years, cancer, described by her son (50-59)	Treatment decisions (2.4, 2.2), Communication (5.3, 5.4)	Ms Bijlsma was given the choice between actively treating her tumour with radiotherapy or to focus on palliation. She chose to receive radiotherapy but was not fully informed about possible side-effects when she made this decision. Her son described: ‘My mother absolutely did not expect it to cause so much pain, which did not reside until her death. (..) The pain made my mother very angry.’ The GP and the oncologist were deterred by her bad mood and did not seem to pay attention to her pain. It was not until she was admitted to a hospice that she received proper pain management.
4	Appropriate	Male, 90-99 years, cancer, heart disease and diabetes, described by his daughter (50-59)	Treatment decisions (2.4), Patient’s wish (4.1).	Despite his age, Mr van Zijl was young at heart. He was scared to die, so he wished to continue active treatment for cancer. His daughter described: ‘The treating physicians have ‘granted’ him one or two surgeries more than they would have done in a comparable person with a lesser will to live.’ His life was prolonged by a few months, in which time he could take care of his wife. Moreover, ‘it gave him the assurance that he had done everything possible to stay alive as long as possible’.

Abbreviations: GP; General practitioner

**Fig. 1:**
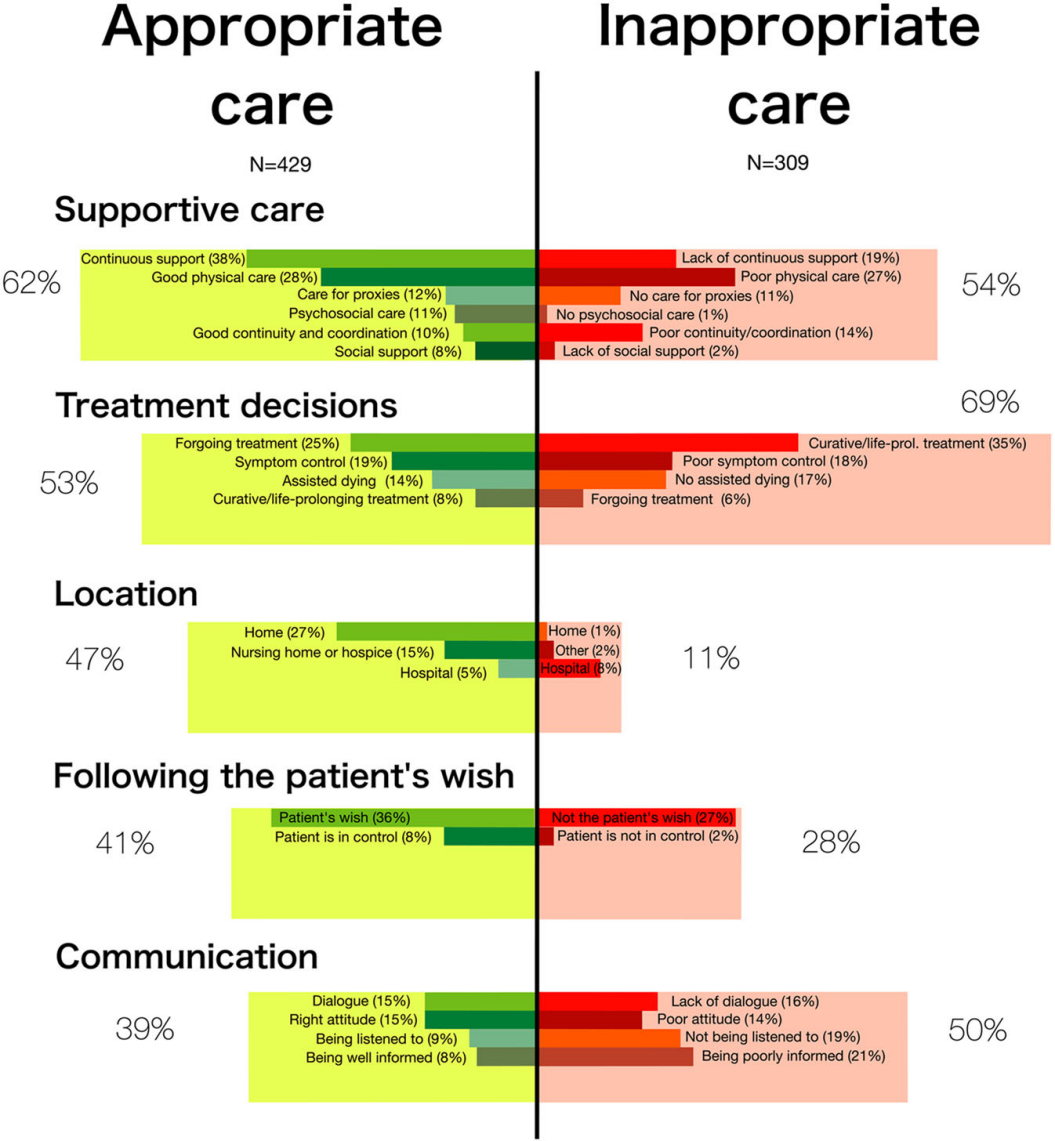
Categories of appropriate (green) and inappropriate (red) care, categorized into five main dimensions. On the left, the frequencies in which the dimensions and categories were described in cases of appropriate care are shown (*n* = 429). On the right, the frequencies in cases of inappropriate care are shown (*n* = 309), directly next to the opposing category in appropriate care. Categories that were mentioned in less than 5 % of cases (both in appropriate care and inappropriate care) are not shown. In appropriate care, these categories are: ‘Other locations’ (1 %, categorized under ‘location’) and ‘other care aspects‘ (1 %, categorized under ‘supportive care’). In inappropriate care, the only category not shown is ‘errors and complications’ (4 %, not categorized). *Ltcf: long-term care facility

In most cases of appropriate care (81 %) and inappropriate care (77 %), more than one dimension was described. Figure 1 shows the prevalence in which dimensions were described as appropriate care (in green) and inappropriate care (in red). The corresponding categories described in at least 5 % of cases are also shown.

In appropriate care, all five dimensions were frequently described (39 %-62 %). Supportive care was the largest dimension in appropriate care (62 %), and mostly concerned ‘continuous support’ (38 %) and ‘good physical care’ (28 %). In inappropriate care, inappropriate treatment decisions (69 %), inadequate supportive care (54 %) and inadequate communication (50 %) were often described. Only treatment decisions and communication played a larger role in inappropriate care than in appropriate care. Especially the treatment decision ‘potentially curative/life-prolonging treatment’ was often described as inappropriate (35 %), while this category was rarely described as appropriate care (8 %). Accordingly, ‘forgoing potentially curative/life-prolonging treatment’ was rarely described as inappropriate (6 %), while it was described in 25 % of appropriate care cases.

### Patient and care characteristics associated with inappropriate care

The cases of appropriate and inappropriate care were compared for patient and care characteristics (Table 4). While cancer was more prevalent in appropriate care, inappropriate care significantly more often concerned patients with other physical diseases and psychiatric disease. Inappropriate care was more often situated in hospital than appropriate care, and less often at home or in a hospice. Physicians were more often involved in inappropriate care, especially clinical specialists, but general practitioners less often played a role in inappropriate care. Nursing staff, patients and relatives contributed to inappropriate care less often than to appropriate care.

**Table 4:** Patient and care characteristics in appropriate and inappropriate care^a^

	Appropriate care	Inappropriate care	*P*-value of a difference^b^
	*n* = 429 %	*n* = 309 %	
Patient characteristics			
Patient gender			
Female	49	53	n.s.
Patient age			
Mean (range)	74 (20-102)	74 (10-101)	n.s.
Diagnosis^c^:			
Cancer^de^	58	47	0.002
Other physical diseases^df^	29	37	0.025
Old age/general decline	21	24	n.s.
Dementia	13	14	n.s.
Psychiatric disease^d^	0.5	3	0.011
None	1	1	n.s.
Care characteristics			
Location of care^c^:			
Home, primary care^d^	57	36	<0.001
Home, specialist outpatient care	17	18	n.s.
Hospital, inpatient department^d^	25	36	0.001
Nursing home or residential home	25	28	n.s.
Hospice^d^	8	3	0.003
Other	3	2	n.s.
Responsible for this care^c^			
Physicians^d^	74	82	0.013
-General practitioners^d^	43	32	0.006
-Clinical specialists^d^	19	37	<0.001
-Elderly care physicians	5	6	n.s.
-Other^d^	1	4	0.015
Nursing staff^d^	59	28	<0.001
Patient and/or relatives^d^	19	8	<0.001
Other^g^	8	8	n.s.

^a^Missing values ranged from 0.0 to 7.1 %

^b^ *P*-value calculated using Fisher’s exact test for dichotomous values and Chi-square test (two-tailed) for categorical variables (cut-off point *p* < 0.05)

^c^One or more answers could be given

^d^Significant difference between appropriate and inappropriate care (*p* < 0.05). n.s.: not significant

^e^Cancer: In appropriate care: 12 % colorectal cancer, 11 % lung cancer, 10 % prostate cancer, 5 % breast cancer, 3 % haematological malignancy, 22 % other, 2 % unknown. In inappropriate care: 7 % colorectal cancer, 9 % lung cancer, 4 % prostate cancer, 5 % breast cancer, 7 % haematological malignancy, 12 % other, 1 % unknown

^f^ Other physical diseases: In appropriate care: 9 % heart disease, 6 % neurological disease, non-CVA, 5 % COPD, 4 % CVA, 9 % other. In 'inappropriate care: 10 % heart disease, 6 % neurological disease, non-CVA, 4 % COPD, 7 % CVA, 16 % other

^g^Other: e.g. chaplains, psychologists and physiotherapists, hospital administration or Health Insurance Company

### Is appropriate care similar for different diseases?

The four main diagnostic groups were cancer, other physical diseases, general decline and dementia. All five dimensions were frequently described in each disease group. Moreover, the relative frequency in which the dimensions were described in appropriate and inappropriate care was mostly similar in the four diagnostic groups. In Table 5 the results of logistic regression analysis for the association between the presence of each of these diagnostic groups and the care dimensions is shown, after correction for gender, age and the other conditions. The presence of cancer did not significantly influence the results, nor did other physical diseases. In case of general decline, inappropriate care less often concerned treatment decisions (OR 0.43). In case of dementia, treatment decisions were less often described both as appropriate care (OR 0.49) and as inappropriate care (OR 0.21). Inappropriate care in dementia more often concerned inadequate supportive care (OR 2.4) and location (OR 3.2), and less often inadequate communication (OR 0.24).

Separate analysis was done for each of the treatment decisions. Category 2.4 (potentially curative/life-prolonging treatment) was described as appropriate more often in other physical diseases (OR 4.0). In case of dementia, participants described category 2.3 (assisted dying) less often both in appropriate and inappropriate care (OR 0.23 and 0.24).

**Table 5:** Association between diagnostic groups^a^ and the prevalence in which the care dimensions were described, in appropriate care (left) and inappropriate care (right): Results of logistic regression analysis. OR and 95 % CI are presented^b^

	Appropriate care				Inappropriate care			
	Cancer	Other physical diseases	General decline	Dementia	Cancer	Other physical diseases	General decline	Dementia
	*n* = 250	*n* = 124	*n* = 91	*n* = 55	*n* = 144	*n* = 114	*n* = 75	*n* = 44
1. Supportive care	-^c^	-	-	-	0.73 (0.36-1.5)	-	-	2.4 (1.1-5.6)
2. Treatment decisions	-	-	-	0.49 (0.25-0.96)	-	-	0.43 (0.21-0.88)	0.21 (0.09-0.48)
2.1 Forgoing treatment	-	-	-	-	-	-	-	-
2.2 Symptom control	-	-	-	-	-	-	-	-
2.3 Assisted dying	-	-	-	0.23 (0.06-0.92)	-	-	-	0.24 (0.06-0.92)
2.4 Curative treatment	-	4.0 (1.4-11.4)	-	0.33 (0.04-2.7)	-	-	-	-
3. Location	-	-	-	-	-	-	-	3.2 (1.1-9.4)
4. Patient’s wish	1.6 (0.84-3.0)	-	-	-	-	-	-	-
5. Communication	-	1.7 (0.97-3.1)	-	-	-	-	-	0.24 (0.06-0.92)

In logistic regression analysis, we tested whether the presence of a diagnostic group was associated with prevalence in which the care dimensions were described. Corrected odds ratios (and 95 % confidence intervals) for the association between the presence of a diagnostic group and the prevalence of the care dimensions (or categories) in appropriate care (left) and inappropriate care (right). The association was corrected for age (categorized in four groups), gender, and presence of the other diagnostic groups. If a significant association was found before correction, which was not significant after correction, this is mentioned in the table

^a^More than one diagnostic group could be present in each case

^b^If the p-value for the association between a diagnostic group and a care dimension or category was <0.10, the odds ratio is presented. Associations with a *p*-value below 0.05 are shown in bold font. – indicates that no association was found (*p*-value >0.10)

^c^Before correction a significant association was found between cancer and supportive care in appropriate care (OR 1.5, 95 % CI 1.01-2.2; after correction OR 1.2, 95 % CI 0.66-2.3)

## Discussion and conclusion

### Discussion

#### What is appropriate care in the last phase of life?

This study shows that patients and relatives interpret appropriate care in the last phase of life as a wide-ranging term, which can refer to supportive care, treatment decisions, location, the role of the patient’s wish and patient-physician communication. These findings are in line with earlier studies, that showed that patients in the last phase of life have multiple and diverse care needs [17, 20–22]. The five dimensions of appropriate care are similar to, but broader than those identified in studies on good palliative care [3, 21]. For instance, the domains described in the NCP Clinical Practice Guidelines for quality palliative care mostly fall under the dimension ‘supportive care’, while focussing little on treatment decisions and location [3]. Apparently, patients and relatives perceive appropriate care in the last phase of life as broader than the presence of good quality palliative care.

#### What is inappropriate care in the last phase of life?

In many cases, inappropriate care could simply be defined as the absence or the opposite of appropriate care. In these cases, care was insufficient to meet the patients’ and relatives’ needs. However, the difference between appropriate care and inappropriate care was not always so clear-cut. While potentially curative or life-prolonging treatment was often described as inappropriate, there were also cases in which it was seen as appropriate. Accordingly, stopping potentially curative or life-prolonging treatment was described as appropriate as well as inappropriate (albeit less often). This illustrates how difficult decisions on starting, continuing or stopping potentially curative of life-prolonging treatment can be in advanced disease. Treatment can be appropriate by giving hope, a chance of prolonging life and it can be the patient’s wish. But in many cases, treatment is more likely to lead to false expectations, side-effects and complications [4]. Physicians need to recognize this risk before starting treatment and take time to discuss this dilemma with their patients.

#### Main risks in care in the last phase of life

An encouraging sign was that the participants described more cases of appropriate care than of inappropriate care. However, improvement is called for, especially in treatment decisions and patient-physician communication. These two dimensions were described in inappropriate care more frequently than in appropriate care, and were identified in other studies as well [23, 24]. Especially potentially curative or life-prolonging treatment was a prevalent category of inappropriate care. Because patient-physician communication also lies at the basis of appropriate decision making, [4, 25–27] we would advise to focus interventions on improving communication in the last phase of life. It can be argued that these interventions should primarily be targeted at clinical specialists, because they often played a role in inappropriate care.

#### Similarities between different conditions/diagnoses

Lynn and Adamson described three trajectories of decline until death; roughly divided into the trajectory in cancer, organ failure and frailty/dementia [18]. Surprisingly, our study showed that descriptions of (in)appropriate care at the end of life were very similar across these diagnostic groups. Only dementia was notably different, with more emphasis on supportive care and location, and less on treatment decisions and communication. The similarity between the other diagnostic groups is in line with other studies describing that care needs at the end of life are quite uniform across different diseases [17, 28]. Despite the similarities, non-cancer patients seemed to receive appropriate care less often than cancer patients. Possibly, the health system around cancer patients is better organized. Another explanation could lie in the disease trajectories described by Lynn and Adamson. In case of cancer, the start of the last phase of life is relatively clearly marked and the phase in which intensified care is needed is generally of short duration (weeks to months). In comparison, providing care to patients with organ failure or frailty could be more difficult, because it is often unclear when the last phase of life has arrived, it can last for years and the patient can unexpectedly decline [18, 29].

#### Strengths and limitations

Using an internet survey enabled us to reach a large number of people who had experienced care at the end of life, without being restricted to a certain disease, location or medical specialty. While previous studies have given overviews of patient needs at the end of life, [1, 3, 21] this study described the care that helped patients and relatives satisfy these needs. This study can be used as a starting point for further research on appropriateness of care.

A major limitation of using an internet survey is the risk of selection bias. Our participants were not randomly sampled. Frail older patients and physically impaired patients were likely to be underrepresented [30]. Still, we were able to gather information about these people through their adult children. Moreover, in the Netherlands 97 % of people have access to internet, and 88 % use internet daily [31]. People from ethnic minorities were underrepresented in our sample (6 %, compared to 21 % in Dutch society [32]). Another limitation was that some participants might have been incomplete in their description of care, and we were unable to ask them to elaborate on their answers. Furthermore, most of the participants in this study were relatives of patients. Therefore, this study might be a reflection of their experiences rather than a reflection of the patients’ experiences. Recall bias might also have led to some distortion. Another limitation concerns the use of retrospective data. This study showed what is seen as (in)appropriate care in hindsight, and could not show how to recognize it beforehand. Finally, because the answers were coded by the researchers, their background might have coloured the results.

#### Conclusion

Although every patient had different needs in the last phase of life, there were similarities in what care patients and relatives considered appropriate or inappropriate. For them, appropriate care in the last phase of life was a broad term and could refer to supportive care, treatment decisions, location of care, the role of the patient’s wish and patient-physician communication. Inappropriate treatment decisions and poor communication were the most important threats to appropriate care. Ideas on appropriate and inappropriate care were remarkably consistent among different conditions, although the extent to which these needs were met was higher in cancer and lower in patients suffering from other physical diseases.

#### Practice implications

We provide physicians, nurses and other caregivers with an overview of important dimensions in end of life care, which should be considered in every patient with advanced disease or at high age. Because every patient has different needs which can change over time, we would advise to repeatedly discuss the patient’s needs concerning each of the identified dimensions. What is appropriate for one patient, may be inappropriate for the other. Especially appropriateness of giving or forgoing potentially life-prolonging treatment can be difficult to assess. When treatment decisions need to be made, physicians should take time to discuss all relevant options with the patient and/or relatives and help them articulate their aims and preferences before decisions are made. These conversations should be had with all patients in the last phase of life, irrespective of diagnosis. To improve communication and decision-making, research and education should aim to improve physicians’ communication skills with patients in the last phase of life.

## Appendix

### Separate analysis for health care workers and members of the Right to Die-NL newsletter

The distribution over the different dimensions was similar for participants who worked in health care and other participants, except for location which was described more often by care workers in the cases of appropriate care (56% compared to 41%), mostly home (36% compared to 20%). Participants recruited through the Right to Die-NL newsletter more often described treatment decisions as appropriate (60% compared to 48%) and as inappropriate (77% compared to 61%). This difference is a reflection of a higher proportion describing assisted dying as appropriate (24% compared to 6%) and describing a refusal or postponing of assisted dying as inappropriate (27% compared to 8%) in this group. In appropriate care, recipients of the Right to Die-NL newsletter put less emphasis on location (41% compared to 52%), especially being home (22% compared to 32%).

## Abbreviations

CI: Confidence interval
COPD: Chronic obstructive pulmonary disease
CVA: Cerebrovascular accident
GP: General practitioner (family care physician)
OR: Odds ratio
